# Light Stimulates the Mouse Adrenal through a Retinohypothalamic Pathway Independent of an Effect on the Clock in the Suprachiasmatic Nucleus

**DOI:** 10.1371/journal.pone.0092959

**Published:** 2014-03-21

**Authors:** Silke Kiessling, Patricia J. Sollars, Gary E. Pickard

**Affiliations:** 1 School of Veterinary Medicine and Biomedical Sciences, University of Nebraska, Lincoln, Nebraska, United States of America; 2 Douglas Mental Health University Institute, McGill University, Montreal, Quebec, Canada; 3 Department of Ophthalmology and Visual Sciences, University of Nebraska Medical Center, Omaha, Nebraska, United States of America; University of Alabama at Birmingham, United States of America

## Abstract

The brain's master circadian pacemaker resides within the hypothalamic suprachiasmatic nucleus (SCN). SCN clock neurons are entrained to the day/night cycle via the retinohypothalamic tract and the SCN provides temporal information to the central nervous system and to peripheral organs that function as secondary oscillators. The SCN clock-cell network is thought to be the hypothalamic link between the retina and descending autonomic circuits to peripheral organs such as the adrenal gland, thereby entraining those organs to the day/night cycle. However, there are at least three different routes or mechanisms by which retinal signals transmitted to the hypothalamus may be conveyed to peripheral organs: 1) via retinal input to SCN clock neurons; 2) via retinal input to non-clock neurons in the SCN; or 3) via retinal input to hypothalamic regions neighboring the SCN. It is very well documented that light-induced responses of the SCN clock (i.e., clock gene expression, neural activity, and behavioral phase shifts) occur primarily during the subjective night. Thus to determine the role of the SCN clock in transmitting photic signals to descending autonomic circuits, we compared the phase dependency of light-evoked responses in the SCN and a peripheral oscillator, the adrenal gland. We observed light-evoked clock gene expression in the mouse adrenal throughout the subjective day and subjective night. Light also induced adrenal corticosterone secretion during both the subjective day and subjective night. The irradiance threshold for light-evoked adrenal responses was greater during the subjective day compared to the subjective night. These results suggest that retinohypothalamic signals may be relayed to the adrenal clock during the subjective day by a retinal pathway or cellular mechanism that is independent of an effect of light on the SCN neural clock network and thus may be important for the temporal integration of physiology and metabolism.

## Introduction

The suprachiasmatic nucleus (SCN) of the anterior hypothalamus contains the primary circadian oscillator in the central nervous system. An interconnected network of autonomous clock cells within the SCN is entrained to the day/night cycle via retinal signals that daily reset clock gene oscillations and neuronal firing of SCN cells [1]. The principal role of the SCN clock is the temporal regulation of physiology, behavior and metabolism which it accomplishes, in part, by orchestrating the phasing of peripheral oscillators located in virtually all tissues and organs of the body [2]. One postulated means by which the SCN regulates peripheral oscillators such as the adrenal gland is by way of descending autonomic circuits. In this manner, the SCN pacemaker may entrain the adrenal molecular clockwork to the light:dark cycle, gating responsiveness to adrenocorticotropic hormone (ACTH) and thereby generating an appropriately phased daily rhythm in adrenal corticosterone secretion [3]–[6].

Niijima and colleagues first reported that light stimulation altered autonomic nerve activity; photic stimulation activated descending sympathetic circuits resulting in an increase in action potential firing in the adrenal branch of the splanchnic nerve [7], [8]. More recently light stimulation has been shown to induce the expression of the core clock genes *Period1 (Per1)* and *Period2 (Per2)* in the adrenal and to stimulate corticosterone secretion via activation of the splanchnic nerve [9]. Importantly, light evokes an increase in corticosterone secretion independent of a detectable increase in plasma ACTH, highlighting the critical role played by the sympathetic splanchnic nerve in conveying retinal signals to the adrenal [9].

Photic signals transmitted from the retina to the adrenal via the splanchnic nerve have been reported to be relayed by way of the SCN clock; SCN lesioning blocks light-induced autonomic nerve activity, gene expression and hormone secretion [7]–[12]. However, there may be at least three different non-mutually exclusive routes or mechanisms by which retinal signals transmitted to the hypothalamus may be conveyed via descending autonomic circuits to peripheral organs: 1) via retinal input to SCN clock neurons; 2) via retinal input to non-clock neurons in the SCN; or 3) via retinal input to hypothalamic regions neighboring the SCN.

Lesions that ablate the SCN also eliminate retinohypothalamic efferent fibers that travel through and/or around the SCN en route to other hypothalamic regions such as the subparaventricular zone (sPVZ) [13]–[20]. The extent to which retinal input to extra-SCN regions in the hypothalamus plays a role in transmitting photic signals to peripheral organs is unknown. The early report of Niijima and coworkers [7] raised the possibility that other hypothalamic areas receiving retinal input “may also be responsible for the alterations in autonomic efferent activities to the adrenal, pancreas and liver after light stimulation”.

The SCN is a heterogeneous structure composed both of clock neurons and neurons that are not rhythmic. Non-clock cells do not fire action potentials or express clock genes rhythmically [21]. Recently, Oster and colleagues [5], reported that wild-type adrenals transplanted into mice rendered arrhythmic following mutations of *Per2* and *Cry1*, retain the ability to entrain to the light:dark cycle. These findings indicate that the retinohypothalamic pathway through which the adrenal clock is entrained is not dependent on a functionally intact molecular clock in the SCN and suggests that retinal signals may be conveyed to descending autonomic circuits by non-clock neurons in the SCN [5].

The effects of light stimulation on the circadian clock are very well characterized and a hallmark of the light-evoked responses in the SCN clock is their circadian phase dependency. The light-evoked transcription of the immediate-early gene *c-fos* and the clock genes *Per1* and *Per2* in the SCN is observed primarily during the subjective night [22]. Light-induced adrenal *Per1* expression and corticosterone secretion are also reported to be circadian phase dependent; *Per1* and corticosterone are induced by light during the subjective night at circadian time (CT)16, CT18 and CT22 but not during the subjective day at CT2, CT4, CT6 or CT10 [9], [23]–[25]. (For nocturnal animals, CT12 is defined as activity onset under constant dark conditions and thus subjective night is defined as approximately CT12-CT24.) These observations are consistent with the interpretation that the SCN oscillator mediates the effects of light on the adrenal. Conversely, light stimulation increases the activity of the splanchnic nerve both during the subjective day and subjective night [7], [8], [12]. Moreover, light-induced increases in adrenal nerve activity during the subjective day (CT6) are irradiance dependent [12]. These latter observations of photic driven increases in splanchnic nerve activity during the subjective day appear to conflict with the interpretation that the oscillatory function of the SCN mediates the effects of light on the adrenal.

The current work assessed the ability of light to induce gene transcription in the adrenal and stimulate corticosterone secretion at multiple time points throughout the subjective day and subjective night. The results suggest that retinohypothalamic signals may bypass the SCN oscillator forming a clock-independent retinal pathway or cellular mechanism with access to descending autonomic circuits.

## Results

### Light-induces *Per2* gene expression in the adrenal throughout the subjective day

**Figure 1:**
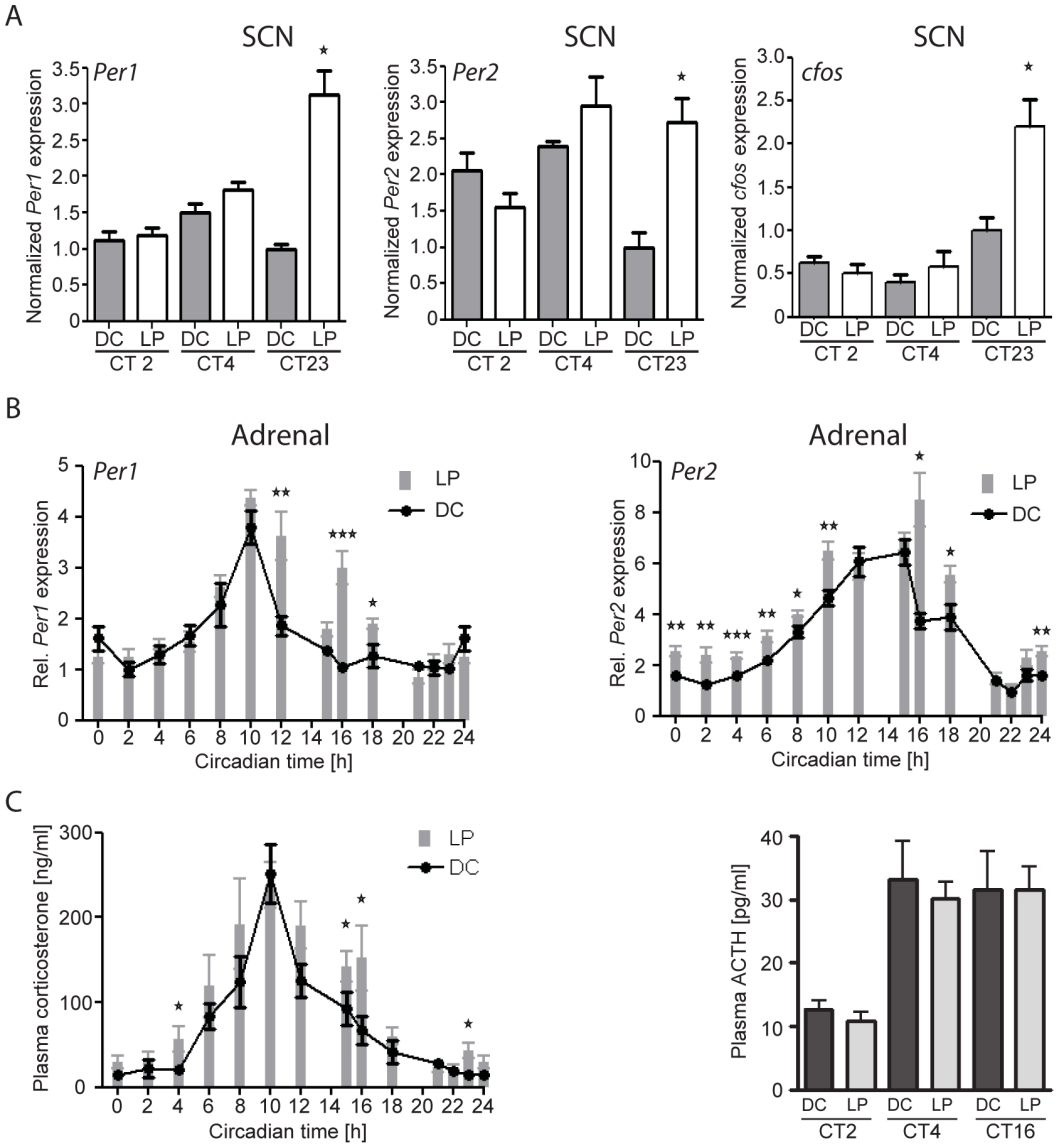
The effect of light on SCN and adrenal gene expression and plasma hormone levels. A: Relative density of *Per1*, *Per2* and *c-fos* expression in the SCN following a light pulse (LP; 30 min at 350 lux) beginning at the indicated circadian time (CT) points compared to dark control (DC) mice. B: Relative expression of *Per1* and *Per2* in the adrenal following a LP compared to DC animals at several CT points throughout the subjective day and subjective night. C: Plasma levels of corticosterone (left) and ACTH (right) following a LP at the indicated CT points. The differences between DC and LP values at each CT point are indicated by asterisks; *p<0.05, **p<0.01, ***p<0.001); n = 4–12 animals/group/CT point.

We reasoned that if photic signals are relayed from the retina to the adrenal via the SCN oscillator, light-induction of gene transcription in the SCN and adrenal would likely be restricted to the same circadian phases. Therefore we examined the effects of light (350 lux, 30 min) on gene induction in the mouse SCN and adrenal at several circadian phases. Light stimulation during the subjective day (CT2 and CT4) produced no change in the transcripts of *Per1, Per2* or *c-fos* in the SCN compared to animals maintained in the dark, whereas during the subjective night (CT23) light-evoked an increase in the SCN expression of all three genes (Fig. 1A). These results are consistent with numerous previous reports indicating that light-induced effects on SCN gene expression and behavior are restricted to the subjective night [22]. In the adrenal, light-induced *Per1* expression was observed only during the subjective night (CT12, CT16 and CT18) (Fig. 1B), in agreement with previous reports [9], [24], [25]. In stark contrast, light induced the expression of *Per2* throughout the subjective day (CT2, CT4, CT6, CT8, CT10) and subjective night (CT16, CT18, CT24) with the exception at CT12-CT15 and CT21-CT23, the peak and nadir, respectively, of the endogenous circadian cycle of *Per2* gene expression in the adrenal (Fig. 1B). Thus, the light-evoked induction profile of *Per2* in the adrenal differs strongly from the light-evoked induction pattern of *Per1, Per2* or *c-fos* in the SCN.

### Light evokes adrenal glucocorticoid secretion during the subjective day and night in an ACTH independent manner

Light stimulation induced gene expression in the adrenal during both the subjective day and night. In the same animals described above we also examined the effects of light stimulation on adrenal corticosterone secretion. In the dark-maintained control animals sampled at multiple phases across the circadian cycle, plasma corticosterone levels exhibited the well-documented circadian rhythm with peak values observed shortly before activity onset at CT10 (Fig. 1C). Light stimulation evoked significant increases in plasma corticosterone levels at CT4, CT15, CT16 and CT23 indicating a functional response of the adrenal to light stimulation during both the subjective day and night. The light-evoked increase in corticosterone levels observed at CT4 stands in contrast to previous reports in which light stimulation was not observed to evoke a significant increase in plasma corticosterone during the subjective day [9], [23]. The light-stimulated increase in corticosterone secretion was not stress-induced as no differences in plasma ACTH levels were observed between light-stimulated and dark-control animals at any of the time points examined (CT2, CT4 and CT16) (Fig. 1C), in agreement with the observation of Ishida et al., [9]. The analysis of adrenal hormone levels after light stimulation indicates that photic signals conveyed to the adrenal via autonomic circuits activate corticosterone synthesis during both the subjective day and subjective night, differing from the responses of the SCN clock to light that are restricted to the subjective night.

### Light induces corticosterone secretion and adrenal Per gene expression during the subjective day in an irradiance-dependent manner

**Figure 2:**
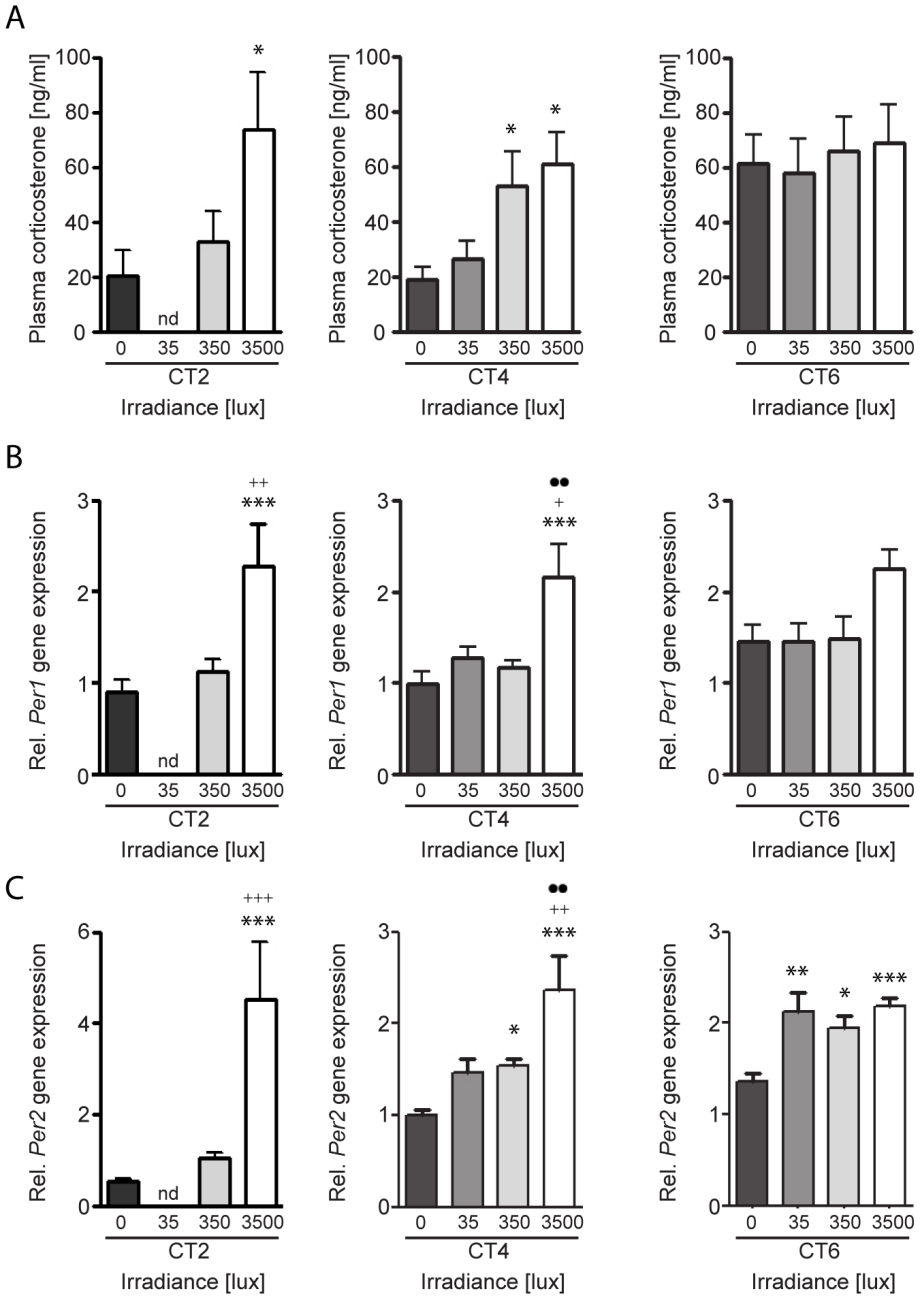
Irradiance dependent effect of light on plasma corticosterone and adrenal *Per1* and *Per2* expression. The effects of a 30(A, top row), adrenal *Per1* mRNA expression (B, middle row), and adrenal *Per2* mRNA expression (C, bottom row). Samples were collected 60 min after the beginning of the light pulse during the subjective day at circadian time (CT) 2, CT4, and CT6. The differences between dark controls (0 lux) and light-pulsed animals are indicated by asterisks (*p<0.05, **p<0.01, ***p<0.001), to 35 lux light-pulsed animals by plus signs (+p<0.05, ++p<0.01) and to 350 lux light-pulsed animals by dots (··p<0.01); One-Way ANOVA, n = 4–9 animals/group.

Although light induced a significant increase in plasma corticosterone at CT4, it was the only time point during the subjective day when light stimulated corticosterone secretion (Fig. 1C). Because the effect of light stimulation on splanchnic nerve activity is both time of day and intensity dependent [12], we re-examined the effects of light stimulation at CT4 and CT6 using 35 lux stimulation and at CT2, CT4 and CT6 using 3500 lux light stimulation to determine if the light-evoked increase in corticosterone secretion and adrenal *Per* gene expression was dependent on irradiance levels. Light stimulation at the lowest intensity examined (35 lux, 30 min) had no effect on plasma corticosterone levels during the subjective day (Fig. 2A). The highest light intensity tested (3500 lux, 30 min) evoked an increase in plasma corticosterone secretion at both CT2 and CT4 (Fig. 2A). The data from the 350 lux light stimulation experiments described above are included in Figure 2. Thus, it appears that the effect of light on plasma corticosterone secretion during the subjective day is both circadian phase and irradiance-dependent.

To explore whether the light-induced changes in plasma corticosterone levels described above are related to light-evoked clock gene expression in the adrenal, *Per1* and *Per2* expression was examined in the same animals (Figs. 2B and 2C). Light stimulation at 35 lux had no effect on *Per1* transcription at CT4 and CT6 or *Per2* transcription at CT4 although a significant increase was noted in *Per2* expression at CT6 (Figs. 2B and 2C). Light stimulation at 350 lux had no effect on *Per1* expression at any of the CTs examined (Figs. 2A–C) whereas this light intensity evoked *Per2* expression at CT4 and CT6 (Fig. 2B). The highest light intensity (3500 lux) evoked *Per1* expression at CT2 and CT4 and *Per2* expression at CT2, CT4 and CT6 (Figs. 2B and 2C). The results collectively indicate that the effects of light on adrenal clock gene expression are circadian phase and irradiance dependent and gene specific and that the threshold for light-evoked adrenal *Per2* induction was lower than that of light-evoked adrenal *Per1* induction. At CTs examined at the nadir of the daily plasma corticosterone levels (CT2 and CT4), light-evoked corticosterone and clock gene expression were strongly related. However, at CT6 during the rising phase of the circadian rhythm of both plasma corticosterone and adrenal *Per1* transcript levels, light was ineffective on altering corticosterone or *Per1* expression at the irradiances tested; *Per2* was inducible at CT6 with all light intensities tested (Table 1).

**Table 1:** The effect of light stimulation during the subjective day on plasma corticosterone and gene expression in the adrenal.

CT	lux	CORT	*Per1*	*Per2*	*TH*	*PNMT*
2	35	nd	nd	nd	nd	nd
	350	-	-	-	-	-
	3500	+	+	+	+	-
4	35	-	-	-	-	-
	350	+	-	+	-	-
	3500	+	+	+	+	+
6	35	-	-	+	-	-
	350	-	-	+	-	-
	3500	-	-	+	-	+

CT  =  circadian time 30 min light pulse was started; lux  =  light intensity in lux; CORT  =  plasma corticosterone; *Per1*  =  *Period1* mRNA expression; *Per2*  =  *Period 2* mRNA expression; *TH*  =  *tyrosine hydroxylase* mRNA expression; *PNMT*  =  *phenylethanolamine N-methyltransferase* mRNA expression; +  =  significant increase vs 0 lux; -  =  not significantly different vs 0 lux dark control; nd  =  not determined.

### Light activation of the adrenal is not mediated via the HPA-axis

**Figure 3:**
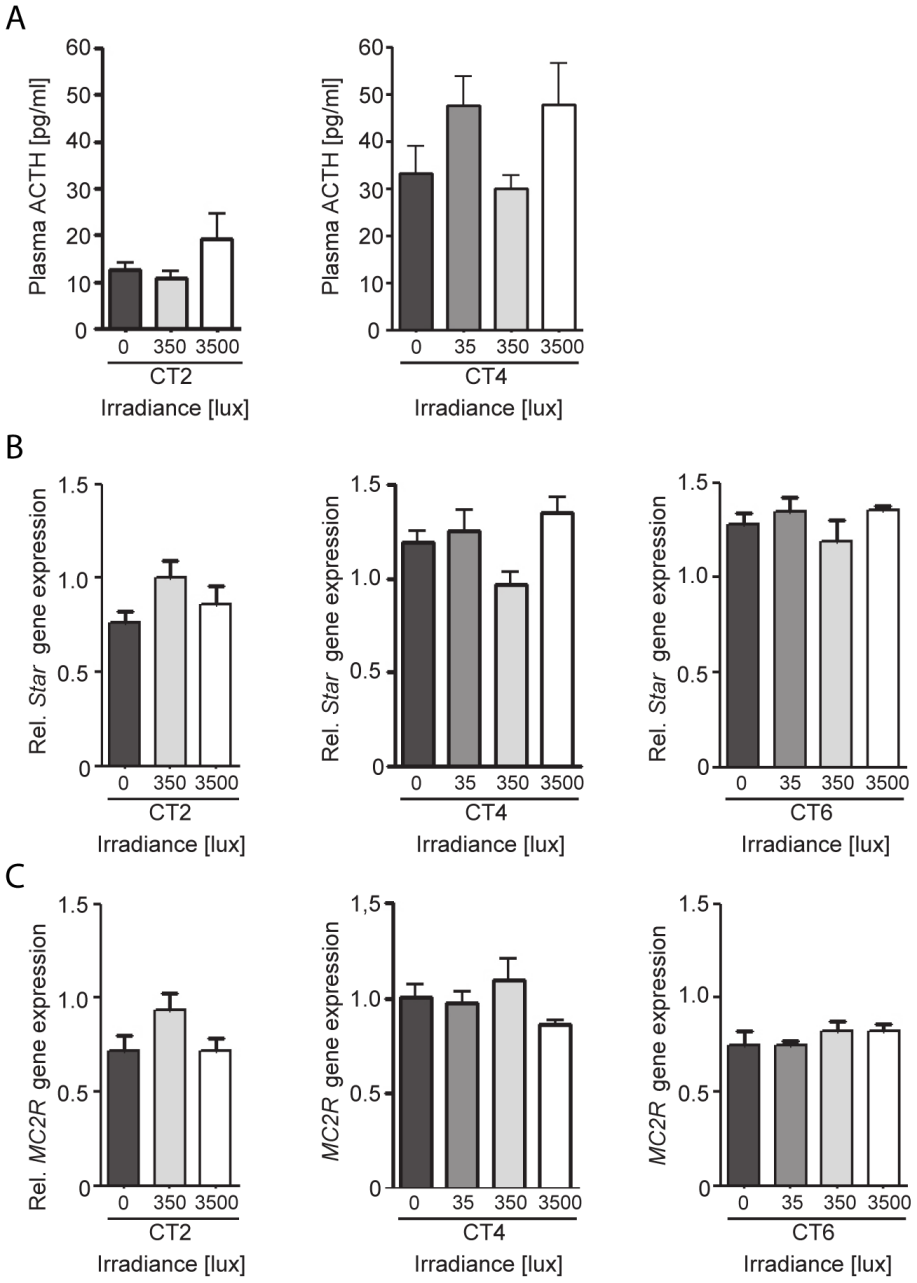
Effect of light on plasma ACTH and adrenal *StAR* and *MC2R* during the subjective day. A: The effect of a 30 min light pulse during the subjective day on plasma ACTH. B: Relative mRNA expression of *StAR* in the adrenal following a 30 min light pulse. C: Relative mRNA expression of *MC2R* in the adrenal following a 30 min light pulse. Samples were collected 60 min after the beginning of the light pulse of either 35, 350 or 3500 lux during the subjective day at circadian time (CT) 2, CT4, and CT6. No significant differences were observed in plasma ACTH, adrenal *StAR* mRNA, or adrenal *MC2R* mRNA expression between light-pulsed and dark control (0 lux) animals at any of the CT points examined; One-Way ANOVA, n = 4–9 animals/group.

Light-stimulated adrenal corticosterone secretion was shown to be independent of an increase in plasma ACTH levels at light intensities of 350 lux (Fig. 1C) and 400 lux [9]. To exclude the possibility that the increase in plasma corticosterone observed after the 3500 lux light stimulation in the current study was mediated by a stress-evoked increase in ACTH, plasma ACTH levels were determined from the same animals in the experiments described above. ACTH levels did not differ significantly between dark-maintained control animals (0 lux) and animals stimulated with 3500 lux light for 30 minutes at CT2 and CT4 (Fig. 3A).

ACTH evokes adrenal corticosterone secretion by interacting with its receptor, (*melanocortin 2 receptor, MC2R*) which up-regulates MC2R mRNA [26] and also induces transcription of *steroidogenic acute regulatory protein* (*StAR)* in the adrenal, which is crucial for the transport of cholesterol to mitochondria where steroid biosynthesis is initiated. *StAR* transcription is considered to be the rate limiting step in adrenal corticosterone biosynthesis [27]–[29]. In the same animals described above, adrenal transcript levels of *MC2R* and *StAR* were determined after light stimulation. No significant differences were observed between dark-maintained and light-stimulated animals at CT2, CT4 or CT6 at any of the three light intensities examined (Figs. 3B and 3C). These results are consistent with the interpretation that activation of the neuroendocrine HPA-axis and ACTH release is not responsible for the observed light-evoked increases in corticosterone secretion noted during the subjective day.

### Adrenal adrenaline synthesis/release may act as a medullary-cortical link for light-induced steroid biosynthesis

**Figure 4:**
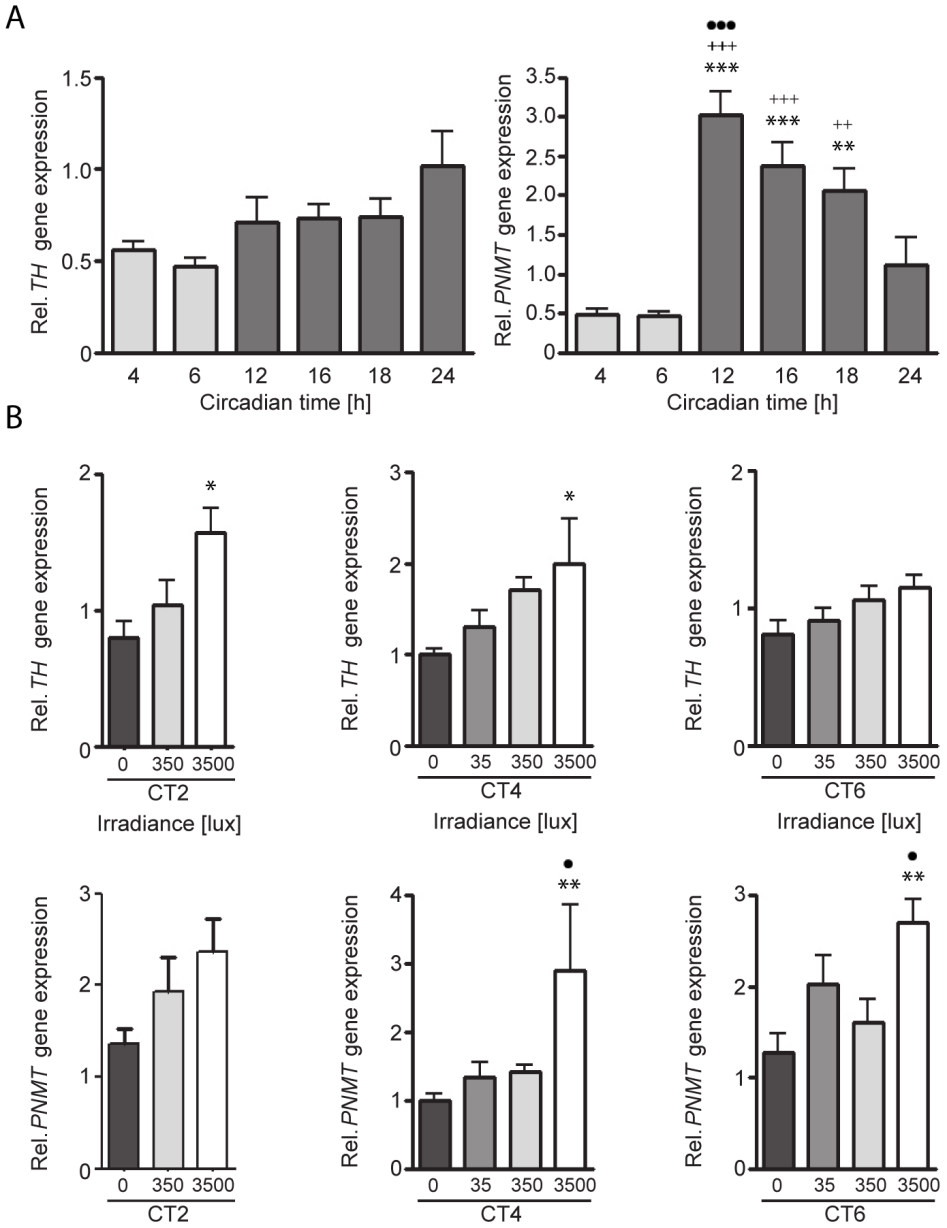
Circadian profiles of adrenal *TH* and *PNMT* and following light stimulation during the subjective day. A: Relative expression of adrenal tyrosine hydroxylase (*TH*) and phenylethanolamine N-methyltransferase (*PNMT*) at several circadian time (CT) points. B: Relative expression of adrenal *TH* and PNMT following a 30 min light-pulse. Samples were collected 60 min after the beginning of the light pulse of either 35, 350 or 3500 lux during the subjective day at circadian time (CT) 2, CT4, and CT6. Significant differences compared to dark controls (0 lux) are indicated by asterisks (*p<0.05, **p<0.01, ***p<0.001), to 35 lux light pulses by plus signs (++p<0.01, +++p<0.001) and to 350 lux light pulses by dots (·p<0.05, ···p<0.001); One-Way ANOVA; n = 4–11 animals/group.

The mechanisms by which light stimulation of the sympathetic input to the adrenal induces clock gene expression and corticosterone synthesis is not known. Light stimulation increases plasma adrenaline (epinephrine) 5 min after light onset (but not noradrenaline or dopamine) and subcutaneous injection of adrenaline induces luciferase luminescence in the adrenal of *Per1-luc* transgenic mice [9]. Thus light-evoked activation of the sympathetic innervation of the adrenal medulla may lead to an increase in catechoamine biosynthesis that acts locally on the adrenal cortex. To examine the potential role of the adrenal medulla on the light-evoked induction of clock genes in the adrenal cortex, we measured transcription of tyrosine hydroxylase (*TH*), the enzyme that catalyzes the rate-limiting step in catecholamine biosynthesis and phenylethanolamine N-methyltransferase (*PMNT*) which converts norepinephrine to epinephrine in the adrenal medulla, at several circadian time points and after light stimulation at CT2, CT4 and CT6. Adrenal *TH* transcript levels did not vary significantly across the circadian day (Fig. 4A) whereas *PMNT* expression was highly elevated during the subjective night (Fig. 4B). Although there was a trend for increasing light intensities to increase *TH* and *PNMT* expression during the subjective day, only light stimulation with the highest intensity tested (3500 lux) significantly increased the transcript levels of *TH* at CT2 and CT4 and *PNMT* at CT4 and CT6 but not at CT2 (Fig. 4B). There is an association between light-induced *TH* and *Per1* induction at the brightest light tested whereas light-evoked corticosterone secretion appears more closely related to *Per2* induction. The mechanism of light-induced activation of adrenal clock genes and corticosterone synthesis remains unclear. The effects of light stimulation on adrenal gene expression are summarized in Table 1.

## Discussion

In the present study, animals housed in complete darkness were exposed to light at different phases throughout the circadian cycle and light-evoked responses of the adrenal gland were measured. The key novel observation is that light presented during the middle of the animal's subjective day stimulated adrenal *Per1* and *Per2* expression and evoked corticosterone secretion. This result was unexpected as the effect of light on the adrenal is thought to be mediated by the SCN clock and the middle of the subjective day is a phase of the circadian cycle when the SCN clock is widely reported to be unresponsive to light stimulation [22]. In agreement with the literature describing phase dependent responses of the SCN clock to light, we also found no light-evoked gene induction in the SCN during the subjective day at CT2 and CT4. Indeed, the characteristic phase dependent light responsiveness of the SCN oscillator is considered a prerequisite for mammals to entrain to the day/night cycle, and this feature of the circadian clock system is a property common to a wide range of organisms [30]. Our findings strongly suggest therefore that photic stimulation of the adrenal during the subjective day is not mediated by the circadian oscillator in the SCN.

Our findings of light-evoked clock gene expression in the adrenal and corticosterone secretion during the subjective day may seem to contrast with previous reports [9], [23]. However, Ishida and co-workers [9] examined light-evoked *Per1* and *Per2* expression in the adrenal at CT16 but only reported on *Per1* expression during the subjective day; the 400 lux light stimulation that was used did not induce *Per1* expression at CT4. We similarly did not observe light-evoked *Per1* expression at CT4 using light at 350 lux although we did observe light-induced *Per2* at CT4 using 350 lux light stimulation. Ishida et al., [9] reported a light-evoked increase in corticosterone secretion at CT16 using 400 lux light stimulation but the light-evoked change in plasma corticosterone at CT4 did not reach statistical significance, perhaps due to the small number of animals stimulated with light (n = 4). These investigators did examine the effects of bright light (2000 lux) on plasma corticosterone but only during the subjective night at CT16 [9]. Mohawk and coworkers [23] examined light-evoked corticosterone secretion at several phases during the subjective day (CT2, CT6, CT10) and subjective night (CT14, CT18, CT22) using 250 lux light stimulation and reported significant increases in plasma corticosterone restricted to the subjective night. Using 350 lux light stimulation, we also did not observe significant light-evoked corticosterone secretion at CT2, CT6, and CT10 whereas we did observe corticosterone levels to be elevated by 350 lux light at CT15, CT16, and CT23 consistent with the findings of Mohawk et al., [23]. Thus, when irradiance levels, time of day and the effects of light on specific clock genes are considered, our findings are entirely consistent with previous reports.

There are at least three possible routes or cellular mechanisms by which light signals transmitted via the retinohypothalamic tract (RHT) may activate descending autonomic circuits: 1) by stimulating clock neurons in the SCN which then transmit photic information to descending autonomic circuits; 2) by stimulating non-clock neurons in the SCN which then transmit photic information to descending autonomic circuits; and 3) by directly stimulating pre-autonomic neurons located in the hypothalamus but outside of the SCN. These three possibilities are not mutually exclusive. Because light stimulation during the subjective day does not alter SCN clock neuron-mediated responses (e.g., *Per1* gene expression and phase shifts of free-running behavioral circadian rhythms) but as shown here, does stimulate adrenal clock gene expression and hormone secretion, our findings do not support an SCN pacemaker-mediated pathway for RHT signals to be relayed to the adrenal.

The numerous reports indicating that light stimulation during the subjective mid-day does not activate molecular components of the circadian clock in SCN neurons or shift the pacemaker to a new phase in the circadian cycle does not however eliminate the possibility that RHT signals can be relayed via SCN clock cells to descending autonomic circuits. SCN clock neurons receiving RHT input during the subjective day may act merely as a conduit for RHT signals to be transmitted to descending autonomic circuits without the retinal signals resetting the molecular clock. For example, pacemaker cells in the marine snail *Bulla gouldiana* respond to light stimulation at all phases of the circadian cycle while light-induced phase shifts of the circadian pacemaker are restricted to the subjective night. Light shifts the pacemaker only during the subjective night because depolarization of clock cells opens calcium channels during the night when these channels are normally closed [31]. Similarly, light stimulation of rodents during the subjective day can substantially boost the firing rate of individual SCN neurons that display rhythmic baseline firing (i.e., clock cells) [32]. However, this increase in activity during the subjective day does not translate into clock gene induction and resetting of the molecular clock apparently because intracellular calcium levels have plateaued at a high level during this phase of the circadian cycle [33]. Light-evoked increases in action potential firing during the subjective day do very little to further increase intracellular calcium levels in SCN clock cells [34]. Therefore after even very bright light stimulation during the subjective day, the increase in clock cell action potential firing rate would have little influence on SCN molecular clock function.

The increase in SCN clock cell firing following light stimulation during the subjective day does not reset clock phase although it may be sufficient to alter the activity of descending autonomic circuits if the light stimulation is of sufficient duration and intensity. The increase in splanchnic nerve activity observed following light stimulation is gradual and the increased activity persists after stimulus termination, peaking in some cases 60–70 min after cessation of light exposure depending on stimulus intensity and duration [7]–[9], [12]. The rise in plasma corticosterone levels following light stimulation parallels the activity of the splanchnic nerve, rising gradually and peaking 30–60 min after light termination [12], [23] and remaining significantly elevated for at least 90 minutes after lights are extinguished [12]. The precise relationship between light intensity and stimulus duration required to evoke adrenal corticosterone secretion at different phases of the circadian cycle remains to be determined. Although light stimulation does not alter the SCN molecular clock during the subjective day, SCN clock cells are a viable route by which light may effect peripheral organs during both the night and day.

An alternate interpretation of our results is that light stimulation of the adrenal during the subjective day is processed by retinal input to the SCN but that the retinorecipient neurons of the SCN that convey photic signals to the adrenal are non-clock neurons and therefore lack phase dependency in their responses. Photic effects on the adrenal in animals lacking core molecular components of the circadian clock are consistent with this interpretation. The SCN circadian clock is non-functional in *Per2/Cry1* double mutant mice, but a light:dark cycle is sufficient to drive rhythmic corticosterone secretion in these animals if they are implanted with a wild-type adrenal [5]. Light pulses induce *Per1* and *Per2* expression in the SCN in *Cry*-deficient mice (*Cry1*/*2* mutant mice) despite behavioral arrhythmicity [35] although SCN neuronal discharge in response to light is strongly reduced in these animals [36], [37]. Mice bearing mutations of the *Per* genes (*Per1*, *Per2* and *Per3*) that result in behavioral arrhythmicity under DD conditions can respond to a 3 h light pulse with transient reorganization of behavioral rhythmicity [38]. Based on findings from *Per1* knockout mice, Pando and colleagues [39] suggested that light may entrain peripheral clocks through a pathway circumventing the SCN.

Photic information may be transmitted via the RHT to descending autonomic circuits in the absence of SCN neurons with a functional molecular clock. These findings are consistent with the hypothesis that non-clock SCN neurons may relay RHT signals to peripheral organs. However, the extent to which SCN clock neurons lacking specific molecular components of the circadian clock are able to convey retinal signals to peripheral targets remains unclear.

Since the initial studies describing a retinohypothalamic projection to the SCN using radioautography [40], [41], it has become increasingly clear from studies applying more sensitive tracing techniques that the RHT terminal field in the hypothalamus extends well beyond the SCN. The medial component of the RHT projects to the sPVZ and the anterior hypothalamic nucleus, in addition to the SCN, whereas the lateral component of the RHT innervates the rostral lateral hypothalamic area [13]–[20], [42], [43]. When SCN ablation is used to evaluate whether the RHT mediates the effects of light on autonomic outflow [7]–[9], [44], retinal afferents to hypothalamic regions outside the SCN (e.g., the sPVZ) are also destroyed. Thus, the demonstration that SCN lesioning blocks the effects of light on autonomic outflow to the adrenal must be interpreted more broadly. The only conclusion that can be drawn from such lesion studies is that destroying the SCN and retinal afferents to the medial hypothalamus blocks the effects of light on autonomic signals to the adrenal.

The specific extra-SCN hypothalamic neurons targeted by retinal efferent fibers to the medial hypothalamus are largely unknown. Retinal ganglion cells innervate gonadotropin releasing-hormone neurons in the primate medial hypothalamus and it has been suggested that this direct retinal link to neuroendocrine cells may provide a pathway independent of but parallel to that of the RHT input to the SCN for photic modulation of hormone release [17]. A similar parallel pathway may exist for the regulation of adrenal clock function and hormone release. Retinal efferents entrain the SCN which in turn regulates rhythmic corticotropin releasing hormone (CRH) secretion and in turn the low amplitude rhythm of ACTH secretion from the anterior pituitary gland. The daily rhythm of ACTH clearly contributes to the daily rhythm in adrenal corticosterone secretion but rhythmic ACTH is not required for the daily corticosterone rhythm [45]. The SCN also drives the daily rhythm in splanchnic nerve activity thereby entraining the adrenal circadian clock [12]. Direct retinal projections to extra-SCN hypothalamic pre-autonomic neurons that may be linked to the adrenal by a multi-synaptic sympathetic circuit via the splanchnic nerve [3], [4] may provide direct entraining signals to the adrenal circadian clock that are independent of but parallel to that of the RHT input to the SCN. The sPVZ is a possible site where pre-autonomic neurons contributing to sympathetic outflow may receive direct retinal input as lesions of the sPVZ eliminate the circadian rhythm of corticosterone secretion [46] and light stimulation during the subjective day (CT6) induces *c-fos* expression in this region [47].

Although activation of the splanchnic nerve is required for light-induced adrenal gene transcription and corticosterone secretion [9], the mechanism(s) by which activation of the splanchnic nerve regulates adrenal clock function remains unclear. Sympathetic innervation of the adrenal gland via the splanchnic nerve is considered to be primarily to the adrenal medulla although a limited number of sympathetic nerve fibers extend from the adrenal medulla into the adrenal cortex, the site of corticosterone synthesis [4]. A link between light stimulation, activation of the splanchnic nerve and adrenaline release from the adrenal medulla was first described by Ishida and colleagues [9]. Plasma adrenaline levels rose quickly after light exposure (400 lux) and exogenous administration of adrenaline increased *Per1-luc* luminescence in the adrenal gland. Electrical stimulation of the splanchnic nerve leads to adrenaline release by the adrenal medulla [48], [49] and adrenaline release by the medulla induces corticosterone synthesis in the adrenal cortex [50]. Thus it appears that light stimulation of adrenal cortical function may act via sympathetic input to the adrenal medulla. However, in the present study, although we found up-regulation of genes involved in catecholamine synthesis after light stimulation, the light-evoked induction of TH and PNMT were not consistently correlated with adrenal clock gene induction after bright light stimulation. Thus the mechanism by which light stimulation of the sympathetic splanchnic nerve activates adrenal clock gene regulation and corticosterone secretion remains unclear.

In this study it appeared that the irradiance threshold for light-induced *Per2* activation in the adrenal was lower than that for *Per1* during the subjective day (Fig. 2). This suggests that *Per2* induction may play a more prominent role in adrenal rhythm generation and clock entrainment. It has also been reported that peak *Per2* expression levels are greater than peak *Per1* levels in the adrenal cortex [51]. Non-redundant roles have been reported for *Per1* and *Per2* in the generation of circadian rhythms in the SCN [52]–[55]. Moreover, the roles of individual clock genes in circadian rhythm generation and period determination appear to be tissue-specific [56]–[60]. Examination of adrenal circadian oscillations *in vitro* using real-time reporters [61] from animals with selective genetic ablation of individual clock genes may reveal non-redundant roles for *Per1 and Per2* in adrenal clock function.

Corticosterone rhythmically produced in the adrenal is a potent synchronizing cue for other peripheral clocks [62]–[66] and therefore functions as an internal synchronizer or ‘body clock’ acting to support the resetting role of the SCN central nervous system clock [67]. However, the data presented in the current study clearly show that RHT signals can regulate adrenal function during the subjective day, when the central clock in the SCN is relatively impervious to the effects of photic stimulation. We have outlined three mechanisms by which light stimulation during the subjective day may alter the activity of descending autonomic circuits to regulate peripheral oscillators: photic stimulation may alter adrenal corticosterone secretion 1) via SCN-clock cells, 2) via SCN non-clock cells, or 3) via cells outside of the SCN, circumventing the SCN clock both functionally and anatomically. Indeed, the effect may be through a combination of these routes, and the current data neither favor nor eliminate any of these options. Because of the important role played by the adrenal as a regulator of circadian physiological and metabolic rhythmicity via its daily rhythm of corticosterone secretion, the possibility exists that parallel and/or redundant mechanisms may have developed to enable this crucial peripheral oscillator to receive information regarding the photic cues provided by the external environment.

## Materials and Methods

### Ethics statement

The use of animals in this study was approved by the University of Nebraska-Lincoln Institutional Animal Care and Use Committee (ID 714) and all experiments were conducted in strict accordance with the recommendations in the Guide for Care and Use of Laboratory Animals of the National Institutes of Health. All efforts were made to minimize suffering.

### Animals

For all experiments male wild-type (C57BL/6J) mice of three to five months of age were used. Mice were housed in small groups of ≤5 animals/cage under 12 h light (100 lux):12 h dark (0 lux) (LD) conditions with food and water available *ad libitum*. For the light pulse (LP) experiments, animals were individually housed in cages for 10 days under LD conditions prior to being released into constant darkness. On the second day of DD, animals were exposed to a 30 min LP of 35, 350, or 3500–4000 lux light intensity at the indicated circadian time points. CT 12 is defined here as the time of light offset in the prior LD cycle. Dark control animals were treated similarly but not exposed to light. The group sizes were as follows: CT0 DC n = 8; LP n = 4; CT2 DC n = 9, LP n = 8; CT4 DC n = 12, LP n = 12; CT6 DC n = 10, LP n = 10; CT8 DC n = 6, LP n = 6; CT10 DC n = 6, LP n = 6; CT12 DC n = 9, LP n = 12; CT15 DC n = 12, LP n = 12; CT16 DC n =  6, LP n = 6; CT18 DC n = 5, LP n = 6; CT21/22 DC n = 4, LP n = 6; CT23 DC n = 5, LP n = 6. Plasma from DC and animals LP with 350 lux at CT2, CT4 and CT6 was assayed for corticosterone levels a second time along with plasma from DC animals and animals given 35 lux and 3500 lux light pulses at those same circadian times so that all the corticosterone values presented in Fig. 2 were determined in a single RIA. This resulted in small differences in the values for these CTs in Figs. 1 and 2.

### Quantitative real-time PCR (qPCR)

Animals (light pulsed or dark controls) were killed by cervical dislocation 60 min after the beginning of the LP. Eyes were removed under a 15 W red safety light prior to tissue dissection. Tissue samples were dissected and stored frozen in RNAlater (Ambion). Total RNA samples from adrenal were isolated using Trizol (Invitrogen) according to the manufactor's protocol. Isolated RNA was dissolved in ddH_2_O and stored at −80°C. The concentration was determined with an ND-1000 NanoDrop) spectrophotometer. cDNA was synthesized using a MultiScript Reverse Transcription Kit including RNase inhibitor (Applied Biosystems) according to the manufactor's protocol with the exception that half of the recommended amount of all reagents per reaction was used and exactly 0.5 μg of RNA was applied. qPCR was performed with the CFX Connect Real-Time PCR Detection System (Bio-Rad) with GoTaq qPCR Mastermix (Promega) according to the manufacturer's protocol. Primer sequences and cycle conditions are detailed in [5], [68]. *Ef1α* was used as standard and single well amplification efficiency estimates and relative quantification of expression levels were performed as described [69]. Statistical analyses were done with GraphPad Prism software (GraphPad Software, San Diego, USA). All data were normalized against the CT-value of *Ef1a* respectively. Data for the induction analyses were normalized against the averaged minimal time point of untreated control animals (CT22). In Fig. 1 the adrenal *Per1* and *Per2* values reported were normalized relative to the averaged value of the DC animals at CT22, the lowest value in the data set and thus set as the normalized value 1. For Figure 2, the same Per1 and Per2 data used in Fig. 1 were used again but here the values were plotted relative to the dark control value at CT4 of each gene. Similar normalization was done for TH and PNMT in Figure 4 allowing for comparison of all genes. Normalization in this manner allows for all the data from each gene in Figure 2 (B & C) to be compared directly.

### In situ hybridization (ISH)

Animals were killed 60 minutes after the beginning of the LP as described above. Tissues were fixed, dehydrated, and paraffin embedded; 8 μm sections were prepared [70] and stored at −80°C. Sections were hybridized with ^35^S-UTP labeled antisense RNA probes for clock gene transcripts [5]. Exposed films were developed and scanned with a flatbed scanner HP Laserjet (Hewlett Packard, Palo Alto, USA). Relative quantification of expression levels was performed by densitometric analysis of autoradiograph films using the Scion Image 4.0 software (Scion Corporation, National Institutes of Health, USA). Three sections per brain were used and for each tissue background was subtracted from adjacent hypothalamic areas on the same slide. Measurements from different animals/experiments were combined for statistical analysis performed with GraphPad Prism software.

### Hormone measurements

#### Corticosterone radioimmunoassay (RIA)

To reduce stress induced effects, animals were transferred to the environmental chamber used for administering the light pulse 2 days prior to the light treatment. Plasma samples were collected 60 min after the light pulse was started. The dark control animals were killed at the same circadian time and in parallel to the treated animals. Plasma samples were stored at −20°C. Plasma was separated using the supernatant of blood collected in Lithium-Heparin tubes after 5 min centrifugation. Plasma corticosterone quantification by RIA (MP Biomedicals) was performed as described [71]. Briefly, for quantification of plasma corticosterone the *ImmuChemTM Double Antibody 125Iod-Radioimmunoassays* (MP Biomedicals, LLC) was used according to the manufactures protocol with some modifications. Plasma samples were diluted 1∶100. All samples were measured in duplicates. The standard curve and specific volumes for all solutions and controls were prepared as described in the manufacture's protocol.

#### ACTH RIA

For quantification of ACTH in plasma an immunoradiometric assay (ACTH IRMA; Cat No. 13002352 130-Kit, DiaSorin, Kiel-Wellsee, Germany) was used. The prepared plasma samples were diluted at 1∶4. All samples were measured in duplicates. The wash solution, standard curve as well as controls were prepared following the manufacture's protocol.

### Data analysis and statistics

Light-induced maxima of gene expression or plasma corticosterone at specific circadian time points were compared with the control values by Mann-Whitney rank sum test even if normality tests and equal variance tests were positive, reflecting the small sample sizes (4 to 12). One way ANOVA was performed to detect statistical differences for more than 2 time points followed by Bonferroni's Multiple Comparison posthoc-test. A statistically significant difference was assumed with p values of less than 0.05. Asterisks, plus marks or circles indicate the significance of differences between values (n.s., not significant; * p≤0.05; ** p≤0.01; *** p<0.001).
